# Unexplained Pleural Effusion Leads to the Revelation of a Malignant Mesothelioma: A Case Report

**DOI:** 10.7759/cureus.24478

**Published:** 2022-04-25

**Authors:** Meriem Rhazari, Othman Moueqqit, Sara Gartini, Sanae El morabit, Safae Diani, Mohammed Aharmim, Afaf Thouil, Hatim Kouismi, Jamal eddine El bourkadi

**Affiliations:** 1 Department of Pulmonology, Mohammed VI University Hospital, Oujda, MAR; 2 Faculty of Medicine and Pharmacy, Mohammed First University, Oujda, MAR; 3 General Medicine, Faculty of Medicine and Pharmacy, Mohammed First University, Oujda, MAR; 4 Department of Pulmonology, Moulay Youssef Hospital, Rabat, MAR; 5 Faculty of Medicine, Mohammed First University, Oujda, MAR

**Keywords:** tuberculosis, asbestos, pleural effusion, malignant mesothelioma, epithelioid mesothelioma

## Abstract

Malignant mesothelioma is a rare and aggressive cancer that usually affects subjects with prior asbestos exposure, a major risk factor that has been widely known as carcinogenic, and its use is now controlled if not banned in many areas of the world. Malignant mesothelioma originates from mesothelial surface cells covering the serous cavities, and the pleura is its most common site. Malignant pleural mesothelioma (MPM) typically presents with pleural effusion and chest wall pain with wide pleural thickening at radiological investigation. Although the histological examination along with immunohistochemistry helps yield the diagnosis, clinicians and experts face many challenges in diagnosing malignant mesothelioma not only due to the rarity of the disease but also due to the similarities that the disease share with other malignancies.

Here, we report a case of a 55-year-old male patient with a history of chronic asbestos work exposure for 12 years who initially presented with unexplained pleural effusion and chest wall pain and was lost to follow-up but came back later with a worsening clinical state. This case is specially presented to raise awareness against cases of unexplained pleural effusion and chest pain.

## Introduction

Malignant mesothelioma is a rare, insidious, and aggressive tumor that has always been described as highly common among patients with a history of chronic asbestos exposure [1,2]. Although asbestos industries have been restricted in many countries, past exposure is responsible for most present cases of the disease as it can take 10-40 years from the initial exposure to develop malignancy [2]. Mesothelioma originates from mesothelial surface cells covering the serous cavities, and the malignant pleural mesothelioma (MPM) is the most common presentation of this malignancy as three main histological subtypes are described: epithelioid, sarcomatoid, and biphasic mesothelioma [2-4]. Mesothelioma can also occur without asbestos exposure as several non-asbestos etiologies are described such as therapeutic irradiation, simian virus-40, and more mineral fibers other than asbestos [5].

Patients with MPM present broad and nonspecific symptoms, such as chest pain, cough, dyspnea, or night sweat, but clinical suspicion should come to light in the presence of a history of professional or occupational asbestos exposure [2]. The radiological findings, the pleural fluid cytology, and the histological results along with the immunohistochemistry studies are key to diagnosing MPM [2]. However, this entity is often complex and difficult to diagnose due to its rarity and histopathological varieties [4].

## Case presentation

**Figure 1:**
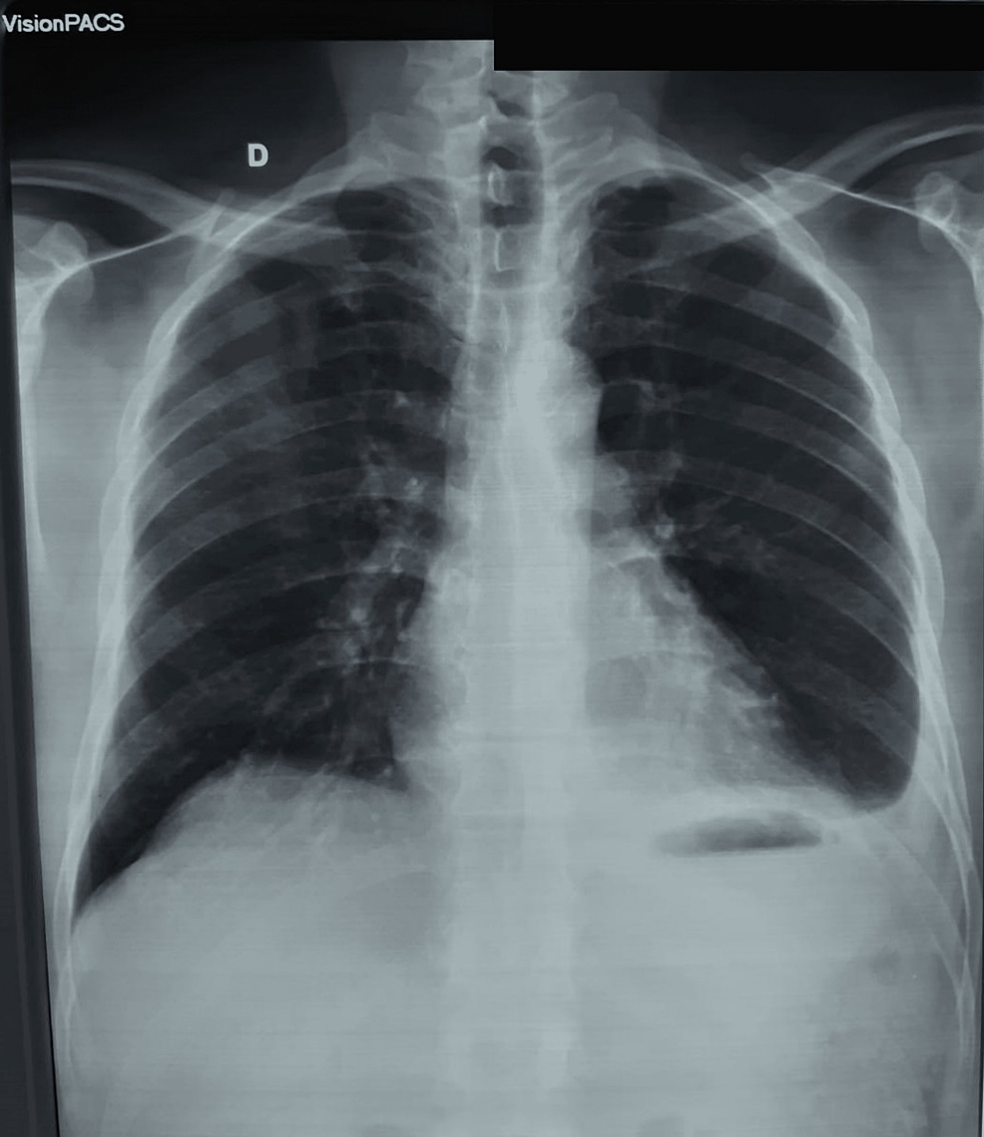
Chest x-ray showing mild unilateral left pleural effusion

**Figure 2:**
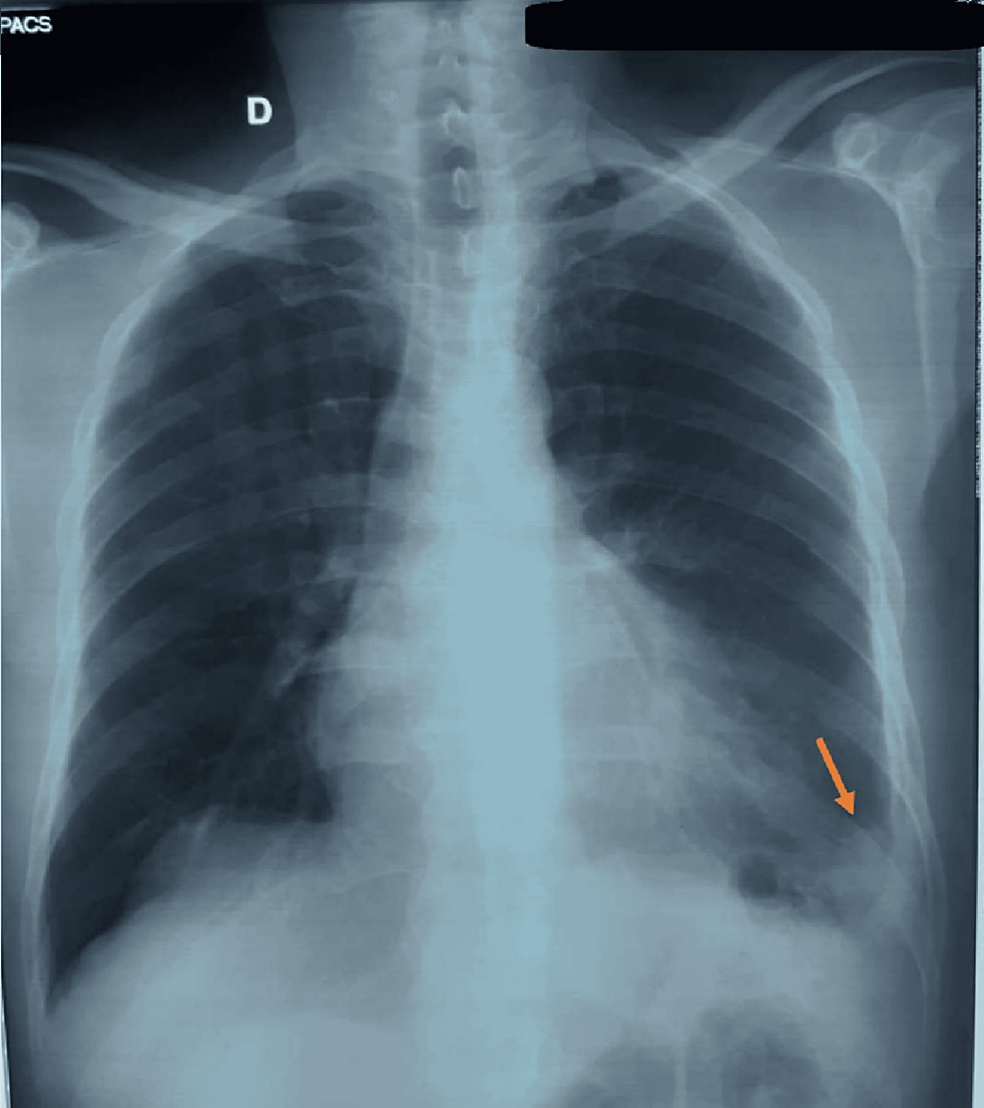
Left basal consolidation with a minimal left pleural effusion

The case of a 55-year-old male patient with a history of chronic asbestos exposure for 12 years during his work for a construction company is presented in this article. His last exposure dates back to 28 years ago. He presented one year ago in another hospital with sharp left chest pain. He had a clear medical history and disclaimed any toxic habits. The patient also denied dyspnea, cough, hemoptysis, and night sweating. At physical examination, breathing sounds in the left lung were decreased and a left pleural effusion was suspected. A chest x-ray confirmed the presence of a low left pleural effusion (Figure 1). At puncture, the liquid was amber and exudate with a total number of white blood cells at 2455/µL, 19% neutrophils, 75% lymphocytes, and 6% monocytes. The bacteriological analysis of the liquid showed no evidence of *Mycobacterium tuberculosis*, no atypical cells were found at cytological analysis, and pleural biopsy turned negative. After four weeks, another chest x-ray was performed, which revealed white infiltrates (Figure 2). Hence, it was decided to treat the patient with antibiotics and keep him under regular follow-up.

**Figure 3:**
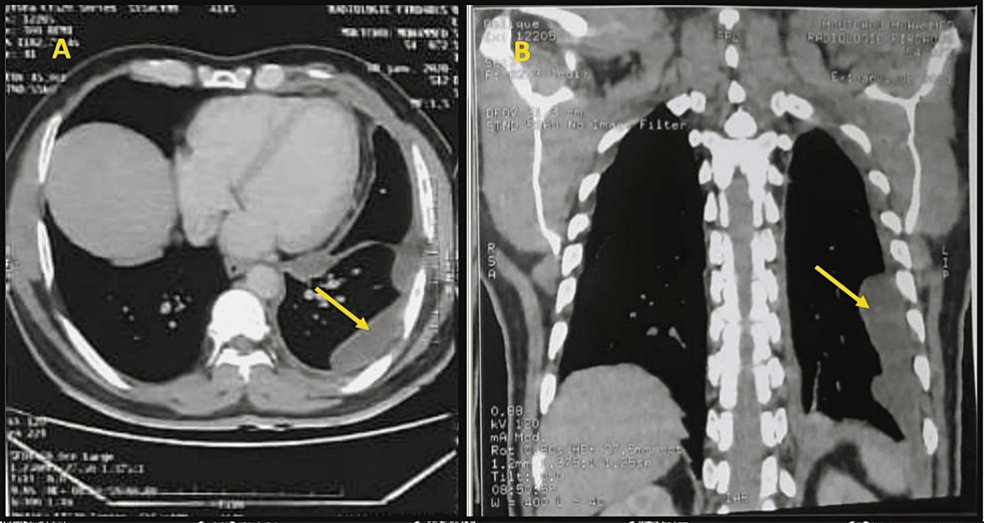
Axial (A) and coronal (B) view of CT chest with contrast showing left nodular thickening of the pleura (yellow arrows)

Unfortunately, the patient was lost to follow-up but came back 11 months later with a sharp and permanent left chest pain with a grade 3 Modified Medical Research Council (mMRC) scale dyspnea and subjective weight loss. At physical examination, he was tachypneic with a respiratory rate of 32 breaths per minute, with decreased breath sounds in the left lung. A chest CT scan (Figure 3) with and without contrast was performed revealing a left nodular thickening of the pleura with pulmonary, sub-, and supradiaphragmatic lymph node metastasis.

**Figure 4:**
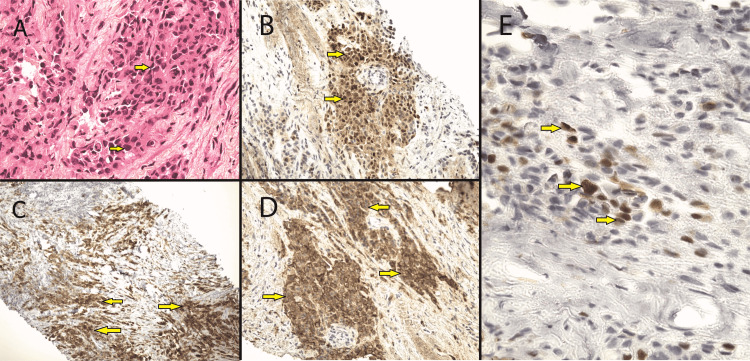
(A) HES coloration, G x 400, proliferation of cuboid cells with medium-abundant acidophilic cytoplasm and hyperchromatic ovoid nuclei. (B) IHC G x 200, calretinin (+). (C) IHC G x 100, D2-40 (+). (D) IHC G x 200, EMA (+). (E) IHC G x 400, WT1 (+). HES: Hematoxylin and eosin stain; IHC: immunohistochemistry; EMA: Epithelial membrane antigen; WT1: Wilms' tumor 1.

A CT-guided trans-parietal biopsy showed large aggregates of malignant cells with eosinophilic intracytoplasmic bodies and irregular nuclei. Immunohistochemistry found a strong reaction of tumoral cells to D2-40, calretinin, Wilms' tumor 1 (WT1), cytokeratin (CK) 5/6, and epithelial membrane antigen (EMA) (Figure 4). The history of asbestos exposure, coupled with the radiological expression, the biopsy, and the immunohistochemistry led to the diagnosis of malignant epithelioid mesothelioma. 

The decision, in this case, was to go for first-line pemetrexed plus carboplatin chemotherapy. The patient reports a remarkable clinical amelioration, and he is under regular follow-up after three months of the treatment.

## Discussion

Malignant mesothelioma is a rare malignancy with an estimated global incidence of 0.30 cases per 100,000 inhabitants in 2020 [6]. And among a variety of plausible causes, asbestos exposure is widely considered the main risk factor as it is reported that up to 90% of MPM in men in Europe and North America are attributable to asbestos exposure [1-6]. The latency interval from the initial causal exposure to the development of the disease varies from 20 to 40 years [7]. A variety of industries have been described as being involved in asbestos consumption, including mining, shipbuilding, manufacturing, and pipe industries. The construction industry remains in lead as it accounted for up to 80% of asbestos consumption between 1999 and 2015 in the United States [7]. Our patient’s history confirms these findings as he reported 12 years of exposure to asbestos working in pipe industries while his last exposure dates back to 28 years before developing the symptoms.

Pleural effusion is the main clinical feature of MPM as most patients present with chest pain, dyspnea, or respiratory distress [2,8]. Constitutional signs such as fatigability and weight loss should be disturbing for every clinician. Initial evaluation consists of a chest x-ray followed by a chest CT scan for MPM. Unilateral pleural effusion, pleural mass, broad pleural thickening, and pleural plaques are the most common radiological findings [2]. Analysis of the pleural fluid cytology via thoracentesis follows, before the histologic study of the pleural mass via thoracoscopic biopsy, along with immunochemistry with selected markers to confirm the diagnosis of malignant mesothelioma [2]. Mesothelial markers such as WT1, calretinin, and D2-40 are mandatory to prove the mesothelial nature of the proliferation and to help eliminate metastatic adenocarcinomas [4].

In our case, pleural effusion and chest wall pain were the earliest clinical signs as fatigue and weight loss appeared later when the patient came back a year later with a grade 3 mMRC scale dyspnea. The initially found pleural effusion by the other doctors remained unexplained before it was accepted as a benign case, and the patient has been put under follow-up before disappearing. In addition to the rarity of the disease, this constitutes a diagnostic challenge as it may cause a delay in diagnosis. Thus, mesothelioma should be suspected with unexplained pleural effusion and chest wall pain as the presence of the constitutional symptoms such as fatigue and weight loss are usually late and associated with a poor prognosis [8]. Furthermore, a diagnosis problem arises from differentiating between epithelioid mesothelioma and carcinomas or other epithelioid tumors that can give metastasis to the pleura, such as lung, breast, ovary, kidney, prostate, gastrointestinal tract, and pancreas cancers [4,8].

Mesothelioma originates from mesothelial surface cells covering the serous cavities [2]. MPM is the most common type, and malignant peritoneal mesothelioma comes second as it accounts for approximately 30% of overall malignant mesotheliomas with an incidence of two to 2.6 cases per million per year [2,9]. Three major histological types were described: epithelioid mesothelioma that represents around 80% of pleural mesotheliomas, sarcomatoid mesothelioma, and biphasic mesothelioma [3,4]. The epithelioid subtype has a better prognosis than the sarcomatoid or biphasic subtype [2,3]. The physiopathology of this rare disease involves multiple explanations. First, the physical features of asbestos fibers determine how deeply they are inhaled to irritate the pleural space. Second is this fiber’s capacity to interfere with different mitosis processes as well as their induced cell damage that generates toxic oxygen radicals, causing DNA and strand break damage [8]. However, observations of familial cases of mesothelioma led to the identification of a mutation in the tumor expressor gene, the BRCA1-associated protein (BAP)-1, that may be involved in increasing the risk of mesothelioma among these families [10]. Other genes such as cyclin-dependent kinase inhibitor 2A (CDKN2A) and neurofibromatosis type 2 gene (NF2) are also frequently involved in the pathogenesis of malignant mesothelioma [4].

As asbestos may be the most important risk factor, a considerable proportion of malignant mesotheliomas was found to be unlikely related to asbestos exposure, especially among women and younger patients (less than 45 years old) [3,5,8]. Other non-asbestos causes of malignant mesothelioma are known in the literature, such as other mineral fibers with similar morphologies and composition, radiation, chronic inflammation, and the genetic predisposition in patients with a mutated BAP-1 gene [3,5,10]. The potential role of simian virus 40 (SV40) in the pathogenesis of malignant mesothelioma is also described but remains open to controversies [8,10].

The management of MPM consists mainly of cisplatin plus pemetrexed chemotherapy when the patient is fit for chemotherapy [10]. Recommendations also suggest adding bevacizumab as a targeted therapy when available to this first-line therapy. Immunotherapy models are promising but need further developments and more results from ongoing phase III trials [10]. Radiotherapy is recommended for palliative purposes, especially for cases of painful sites of MPM [10]. On the other hand, pleurodesis via thoracoscopy is strongly recommended for recurrent MPM cases, while radical surgery is still lacking evidence from randomized controlled clinical trials [10].

The prognosis of malignant mesothelioma remains poor with a median survival of nine to 12 months |6]. It has also been described that a bad prognosis is also associated with weak performance status, being male, high white blood cell count, chest wall pain, and the sarcomatoid histological type [8]. Many countries have tried long ago to either ban or control the use of asbestos as awareness increased about its risk in developing malignancies [2,6,11]. However, malignant mesothelioma is considered a world health issue, not only due to its poor prognosis but also due to its increasing incidence despite the wide regulatory actions taken to decrease its use [7,10]. This can be attributed to the fact that although the development of the disease is the result of prior exposure of 20-40 years, exposure to asbestos fibers is still possible during maintenance activities, remediation, and demolition of old structures and buildings containing pre-existing asbestos fibers [7]. Furthermore, family members of workers involved in activities at risk of asbestos exposure also have the possibility of being exposed [7].

This case study has one limitation. A CT scan was mandatory after thoracentesis when an infiltrative mass was found, especially with high lymphocyte counts, which could help in diagnosing this condition earlier. However, it was accepted that an infection explains the radiological appearance, and antibiotics were started.

## Conclusions

Malignant mesothelioma is a rare and deadly cancer. It mainly affects the subjects with a chronic history of asbestos exposure with a time interval of 20-40 years between exposure and appearance of malignancy. Despite the attempts by many countries to control the consumption of asbestos in commercial and industrial activities, malignant mesothelioma remains a serious threat with a poor prognosis, and awareness should be increased in treating subjects with unexplained pleural effusion and chest wall pain, with or without a history of asbestos exposure.
